# Reducing slab boundary artifacts in three‐dimensional multislab diffusion MRI using nonlinear inversion for slab profile encoding (NPEN)

**DOI:** 10.1002/mrm.26027

**Published:** 2015-10-28

**Authors:** Wenchuan Wu, Peter J. Koopmans, Robert Frost, Karla L. Miller

**Affiliations:** ^1^ FMRIB Centre, Nuffield Department of Clinical Neurosciences, University of OxfordOxfordUnited Kingdom

**Keywords:** multi‐slab, boundary artifacts, 3D diffusion MRI, nonlinear inversion

## Abstract

**Purpose:**

To propose a method to reduce the slab boundary artifacts in three‐dimensional multislab diffusion MRI.

**Methods:**

Bloch simulation is used to investigate the effects of multiple factors on slab boundary artifacts, including characterization of residual errors on diffusion quantification. A nonlinear inversion method is proposed to simultaneously estimate the slab profile and the underlying (corrected) image.

**Results:**

Correction results of numerical phantom and in vivo data demonstrate that the method can effectively remove slab boundary artifacts for diffusion data. Notably, the nonlinear inversion is also successful at short TR, a regimen where previously proposed methods (slab profile encoding and weighted average) retain residual artifacts in both diffusion‐weighted images and diffusion metrics (mean diffusion coefficient and fractional anisotropy).

**Conclusion:**

The nonlinear inversion for removing slab boundary artifacts provides improvements over existing methods, particularly at the short TRs required to maximize SNR efficiency. Magn Reson Med 76:1183–1195, 2016. © 2015 The Authors. Magnetic Resonance in Medicine published by Wiley Periodicals, Inc. on behalf of International Society for Magnetic Resonance in Medicine. This is an open access article under the terms of the Creative Commons Attribution License, which permits use, distribution and reproduction in any medium, provided the original work is properly cited.

## INTRODUCTION

Improvements in spatial resolution for diffusion MRI (dMRI) of the brain can provide the ability to resolve small structures, enabling investigation of finer neuroanatomical features and detailed white matter fiber architecture. Although two‐dimensional (2D) acquisition can increase the in‐plane resolution significantly 1, 2, 3, 4, 5, 6, 7, 8, this approach faces limitations when small isotropic voxels are required. Most importantly, 2D methods couple the repetition time (TR) to the number of slices, such that whole brain coverage necessitates long TR (typically 6–10 s). In the brain, signal‐to‐noise ratio (SNR) efficiency of spin echo sequences is maximized when TR = 1–2 s, meaning that 2D acquisitions are far from optimal for even modest resolution 9. This effect becomes acute at high resolutions due to the competing needs for a large number of slices to cover the brain (requiring longer TR) and high SNR efficiency to support reduced voxel volume (favoring shorter TR). Although simultaneous multislice methods mitigate these issues to some degree, the coupling between the number of slices and the TR will remain an issue, particularly at high resolutions.

Three‐dimensional (3D) acquisitions remove this coupling by exciting the entire volume and defining voxels based on 3D k‐space, acquiring the entire image over multiple readouts (multiple shots). Nevertheless, the use of 3D acquisitions for dMRI faces several limitations. Most importantly, in order to produce image volumes reasonably quickly, 3D dMRI requires very short TR (∼100 ms), which is also very SNR‐inefficient 9. Another challenge comes from the motion‐induced phase errors, which can degrade image quality in multishot acquisitions if not addressed properly. Applying a full 3D nonlinear phase correction method 4 requires time‐consuming 3D phase navigators, which would significantly diminish the scan efficiency. Acquisition of 3D phase navigators could be avoided by using approaches such as driven equilibrium diffusion preparation 10, acquisition with a small field of view (FOV) 11, 12, 13, cardiac gating 11, 14, 15, and estimation of 3D phase errors using data from multiple shots 16, 17. However, these methods still retain residual artifacts, especially when diffusion encoding is along the superior–inferior direction, the direction in which brain pulsation is most significant 18, 19, 20.

To overcome the limitations faced by 2D and 3D acquisition, 3D multislab acquisition has been proposed for high‐resolution dMRI 9, 15, 21. In 3D multislab acquisition, the whole volume is divided into multiple smaller slabs, each of which is excited and encoded separately (i.e., each slab is in effect a reduced‐FOV volume along the slice direction). As a 3D imaging method, it can easily achieve thin slices by increasing phase encoding lines in the slice direction. With proper choice of slab thickness, motion‐induced 3D phase errors in each slab can be well approximated by a 2D navigator 21, 22. This hybrid method partly removes the coupling between number of slices and TR: although TR is proportional to the number of slabs, the slice definition within each slab is accomplished using 3D k‐space encoding. The number of slabs required to cover a given volume is much less than the number of slices and is primarily dictated by the thickest slab that can be phase‐corrected with a 2D navigator. In practice, the whole brain can be covered in 12–14 slabs, for which the TR is highly compatible with maximizing SNR efficiency.

**Figure 1:**
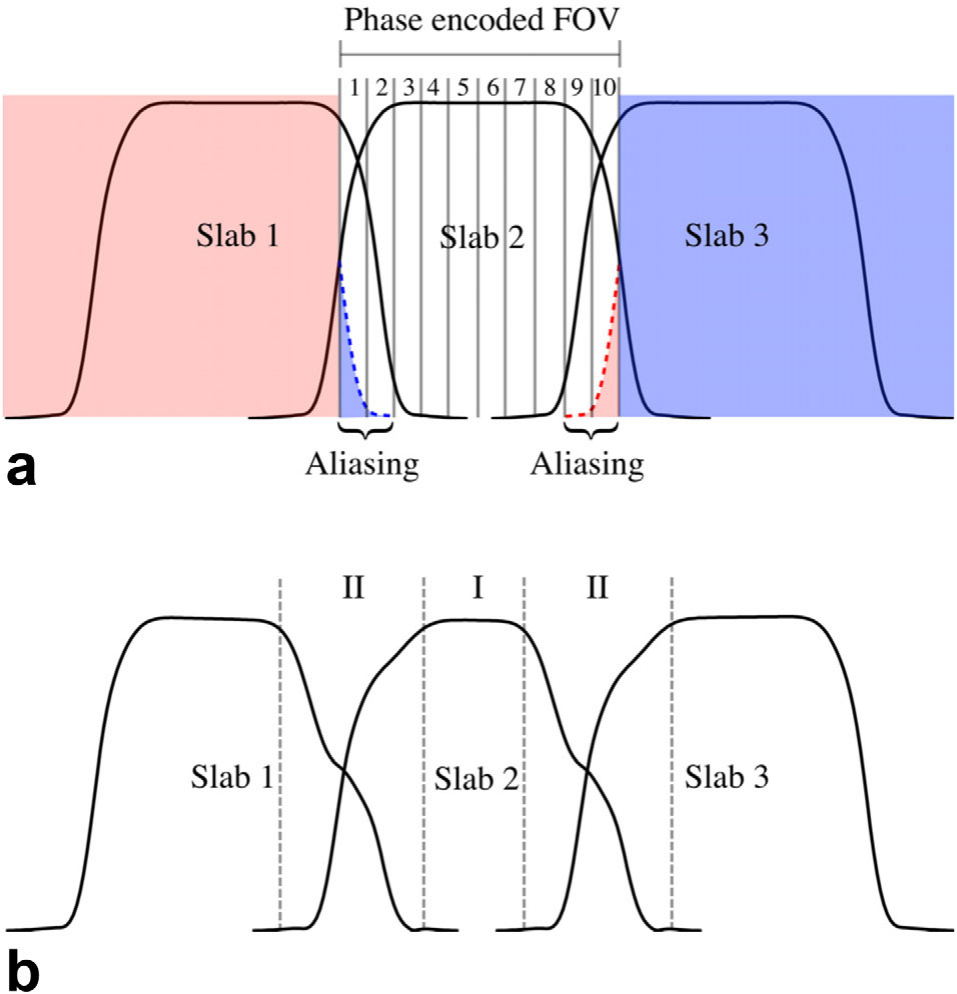
(**a**) Illustration of slab aliasing. The multislab acquisition shown here excites three slabs with 10 $k_{z}$ phase‐encodes for each slab. Extension of the slab profile is wider than the phase encoded FOV (also the predefined slab thickness), leading to aliasing artifacts. (**b**) Illustration of slab crosstalk effect. The slab profile extends to its adjacent slabs, exacerbating T1 saturation effects at slab boundaries where adjacent slabs overlap. In this example with three‐slab acquisition, magnetization at the slab center (region I) is excited every TR, whereas magnetization at slab boundary (region II) is excited with intervals of 2/3TR, 1/3TR, 2/3TR, 1/3TR…, leading to decreased signal at slab boundaries and asymmetry in the shapes. Slab profiles were generated using Bloch simulation with TR = 20 s (a) and 2 s (b), respectively, and interleaved acquisition order. Details of other simulation parameters are described in the Methods section.

A remaining challenge for 3D multislab acquisition is the slab boundary artifact 23, which stems from the shape of the RF profile and appears as periodic signal modulation in the slice direction. Ideally, the RF profile of a slab would have rectangular shape and the width of the slab thickness, but this requires RF pulses to be infinitely long. In reality, RF pulses are truncated, resulting in a nonrectangular RF profile, including magnitude variation inside the slab, transition bands, and side lobes. Therefore, the slab edges have lower signal magnitude than the slab center. The transition bands and side lobes can extend to the neighboring slabs, introducing slab crosstalk and aliasing (Fig. 1).

Several methods have been proposed to reduce the slab boundary artifacts. The most widely used methods include oversampling in the slice direction, deliberate overlapping of the adjacent slabs 23, 24, 25, separating acquisitions for odd/even slabs 15, and applying a (weighted) slab combination at the slab boundary region 9, 15, 21, 23, 24, 25, 26. These methods work well in dMRI if long TRs (∼4 s) are used together with large amounts of slab overlap, but they exhibit residual artifacts in shorter TR acquisitions 27. A variation of the overlapping method shifts the slab by one slice position once all *k_z_* phase encoding steps for a designated subset of *k_y_* phase encoding steps are acquired to transform the signal modulation in the slice direction to the *k_y_* phase encoding direction, using signal demodulation to reduce ghosting artifacts 28, 29. This method has also been demonstrated in non‐Cartesian sequences 30, 31, 32. However, if the signal modulation is not corrected properly, this method may retain ghosting artifacts. Another drawback to these methods is the reduction of scan efficiency due to oversampling, overlapping, or discarding some slices. A histogram‐matching method was proposed to correct the slab boundary artifacts without increasing scan time, which assumes the slab boundary artifact is a global intensity variation in the slice direction 33. However, when this assumption is not valid, residual artifacts remain after correction. Recently, a slab profile encoding (PEN) method was proposed to minimize slab boundary artifacts with a minor increase in scan time 34. PEN treats multislab acquisition as a linear encoding problem, which can be solved by linear inversion approaches (e.g., least‐squares/pseudo‐inverse) as used in SENSE 35, with the slab profiles effectively substituting coil sensitivity profiles. With slab profiles estimated from calibration data and long TRs (∼4 s), PEN can effectively correct aliasing, but some residual artifacts remain due to slab crosstalk, especially at shorter TRs required to maximize SNR efficiency.

We present a method to minimize the slab boundary artifacts for 3D multislab dMRI with SNR‐optimal TRs (∼2 s). We first present detailed simulations demonstrating that the slab boundary artifact is a combination of slab crosstalk and aliasing. Whereas aliasing has been explained and addressed in PEN 34, slab crosstalk effects have not been studied in detail. We characterize the effect of multiple factors (acquisition scheme, TR, T1, B0 inhomogeneity) on slab crosstalk and how it may alter the measurement of diffusion metrics, despite signal normalization by b = 0 images as part of quantification. We then introduce a nonlinear inversion extension of PEN, which we call NPEN, where the slab profile encoding is formulated as a nonlinear optimization. Two constraints are added, which enforce in‐plane smoothness on the slab profile and suppress frequency components corresponding to interslab distance. Results from simulation and in vivo data demonstrate the proposed method can effectively reduce the slab boundary artifacts for 3D multislab dMRI with a short TR of 2 s, a regimen in which weighted average (WA) and PEN reconstructions contain significant residual slab boundary artifacts.

## METHODS

### Bloch Simulations

This section is focused on simulation of slab crosstalk effect, including its causes and how it affects diffusion measurements. These results are crucial motivation for the correction method presented in the following section, where aliasing effects are also discussed.

#### Slab Crosstalk in Multislab Imaging

Figure 1b depicts a Bloch simulation of a multislab acquisition, including both RF profile and T1 saturation effects. The central part of each slab is not overlapped with adjacent slabs, so the magnetization in this region experiences an RF pulse every TR. At slab boundaries, the magnetization is excited every ∼TR/2, but with both the timing and flip angle varying for the target and adjacent slab excitations, which exacerbates T1 saturation and results in lower signal. This effect is called slab crosstalk.

The precise nature of slab crosstalk is affected by a range of factors, including RF profiles, excitation order, TR, T1, and B0 inhomogeneity. To obtain a quantitative understanding about how these factors affect the slab crosstalk, we performed Bloch simulations of the spin echo sequence used in this work 7. The simulations used 600 isochromats in each voxel, which are summed to estimate the MR signal. We simulated a range of TRs and acquisition schemes (sequential and interleaved slab ordering), the three major types of tissue in human brain (white matter [WM], gray matter [GM], and cerebrospinal fluid [CSF]), and a range of off‐resonance frequencies. Simulations were matched to the pulse sequence design used for the in vivo experiments described in later sections. The sequence used 90° and 180° RF pulses designed with the SLR algorithm 36 with a time‐bandwidth product of 20 and 8, respectively, which were chosen to achieve a sharp composite RF profile. The RF pulse waveforms are plotted in Supporting Figure S1. The duration for excitation pulse and refocusing pulse were 7.18 ms and 10.24 ms. The echo times for the first and second echo (phase navigator) were 78 ms and 122 ms. T1 and T2 values for 3T were assumed: WM, 840/70 ms; GM, 1320/110 ms; and CSF, 3000/2000 ms 37, 38, 39. In all simulations, a 15‐mm slab thickness was chosen, because this thickness allows whole brain coverage with TR = 2 s while the slabs remain thin enough to be corrected with 2D navigators 21. Each slab contained 10 slices, 20% of which were overlapped with slices from adjacent slabs to favor PEN and NPEN reconstruction (i.e., two slices on each side).

**Figure 2:**
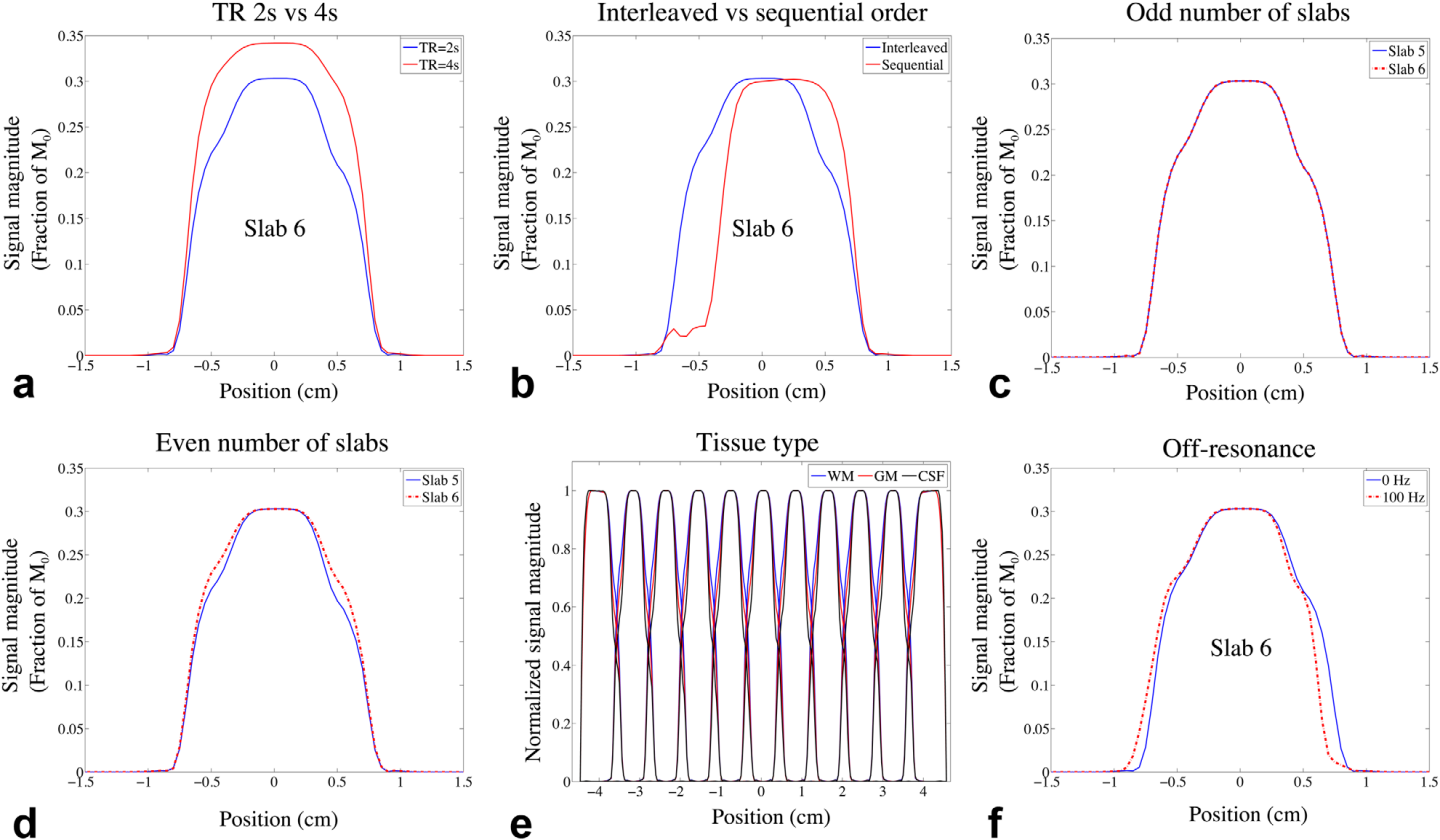
Results of Bloch simulation. (**a**–**d**) Comparison of signal profiles of WM generated from Bloch simulation using different TRs, acquisition schemes, and number of slabs. (a) 11 slabs, interleaved acquisition with TR = 2 s (blue) and TR = 4 s (red). (b) TR = 2 s, 11 slabs with interleaved acquisition (blue) and sequential acquisition (red). (c) TR = 2 s, interleaved acquisition with 11 slabs; the signal profiles of slab 5 (blue) and slab 6 (red) are overlaid for better comparison. (d) TR = 2 s, interleaved acquisition with 12 slabs; the signal profiles of slab 5 (blue) and slab 6 (red) are overlaid. The effects of TR, acquisition scheme, and number of slabs on GM and CSF are similar to WM and are not shown here. (**e**) Slab profiles for WM, GM, and CSF, which were generated by normalizing the MR signal profile with the signal magnitude of the center slice. (**f**) Centered signal profiles simulated with 0 Hz and 100 Hz off‐resonance frequencies.

The effects of TR, acquisition schemes, and B0 inhomogeneity on slab crosstalk are demonstrated in Figure 2. Figure 2a compares the simulated signal profile with different TR, showing reduced saturation effect at slab boundaries with longer TR, although in practice this comes at the significant cost of SNR efficiency. Figure 2b compares acquisitions with different slab ordering schemes. Sequential acquisition suffers from substantial signal loss at one side of the slab, while interleaved acquisition shows high signal intensity at both sides of the slab. Interleaved acquisition with an odd number of slabs produces almost identical signal profile for the odd and even slabs (Fig. 2c), whereas in the acquisition with an even number of slabs, the signal profile for the odd slab varies from the even slab (Fig. 2d). In the following simulations and in vivo scans, interleaved acquisition with an odd number of slabs was used to achieve homogeneous slab profiles with a well‐defined periodicity, which facilitates NPEN reconstruction (described below). Figure 2e compares the slab profiles for WM, GM, and CSF, each normalized by the signal magnitude of the center slice to emphasize differences in shape due to T1 saturation effects.

To simulate the effects of B0 inhomogeneity, Bloch simulation was performed with off‐resonance frequencies starting from −250 Hz to 250 Hz in 5‐Hz increments. The signal profile of one slab in WM is shown in Supporting Figure S2. The profile not only shifts but also becomes distorted, resulting in more signal loss at the slab boundaries (Fig. 2f). The distortion is caused by the difference in bandwidths of the excitation and refocusing pulses. It should be noted that a constant off‐resonance frequency for each slab is assumed in the B0 inhomogeneity simulation, but in reality, the off‐resonance frequencies are spatially varying, which can distort the signal profile even with matched bandwidths of the excitation and refocusing pulses.

#### Propagation of Slab Crosstalk into Diffusion Quantification

The practical importance of variations in signal intensity at slab boundaries will depend on whether these artifacts alter diffusion quantification, based on signal attenuation relative to a non–diffusion‐weighted (b = 0) reference. Multiplicative slab artifacts that attenuate diffusion‐weighted and unweighted signals equally would be removed when normalizing diffusion‐weighted images by the b = 0 reference. In this case, the only effect on diffusion quantification would be SNR reduction at slab boundaries. Conversely, non‐multiplicative effects could create a more significant artifact by introducing bias in diffusion metrics at different regions of slab profiles. For example, mean diffusivity (MD) or fractional anisotropy (FA) could exhibit a consistent bias at slab boundaries. One potential cause of non‐multiplicative attenuation is “partial volume” effects, in which both relaxation and diffusion alter the signal to create a more complicated signal dependence than either effect on its own.

To demonstrate non‐multiplicative effects in diffusion metrics due to partial volume, we simulated a two‐compartment diffusion‐weighted signal corresponding to WM/GM and WM/CSF, respectively 40:

(1) $$M\left(b,v\right)=M_{0}\sum_{i=1}^{2}f_{i}\;\exp\left(-b{v^{T}D}_{i}v\right)\;\left(\sum_{i=1}^{2}f_{i}=1\right).$$

Here, *f_i_* and *D_i_* (*i* = 1,2) are the volume fractions and diffusion tensors for the two compartments and *v* is the diffusion gradient vector. The b = 0 signal *M*_0_ is calculated using Bloch simulations and the diffusion‐weighted signal *M* is subsequently calculated from the partial volume model. The diffusion tensor eigenvalues [*λ*_1_, *λ*_2_, *λ*_3_] for WM [1.4,0.35,0.35] (FA = 0.707), GM [0.7,0.7,0.7] and CSF [3.0,3.0,3.0] were used 40. A set of six noncollinear directions was assumed for *v*. A wide range of b value (1 – 10,000 s/mm^2^) was investigated. We calculated MD and FA for a simulated voxel with a given partial volume, considering these as broadly representative of bias in diffusion metrics (although specific model parameters will vary in their sensitivity to slab boundary artifacts). The goal of these simulations was to identify any consistent bias in diffusion metrics for the slab boundary relative to the slab center. A relative error *ε_r_* was calculated to analyze the slab crosstalk effect on MD (*ε_r_* = |*θ_c_*−*θ_b_*|/*θ_c_*, where *θ_c_* and *θ_b_* are the MD values at slab center and boundary). As the FA values of CSF and GM approach zero when their fractions go to 1, leading to inflated measure of relative errors, we calculated the absolute FA errors between slab center and boundary instead.

**Figure 3:**
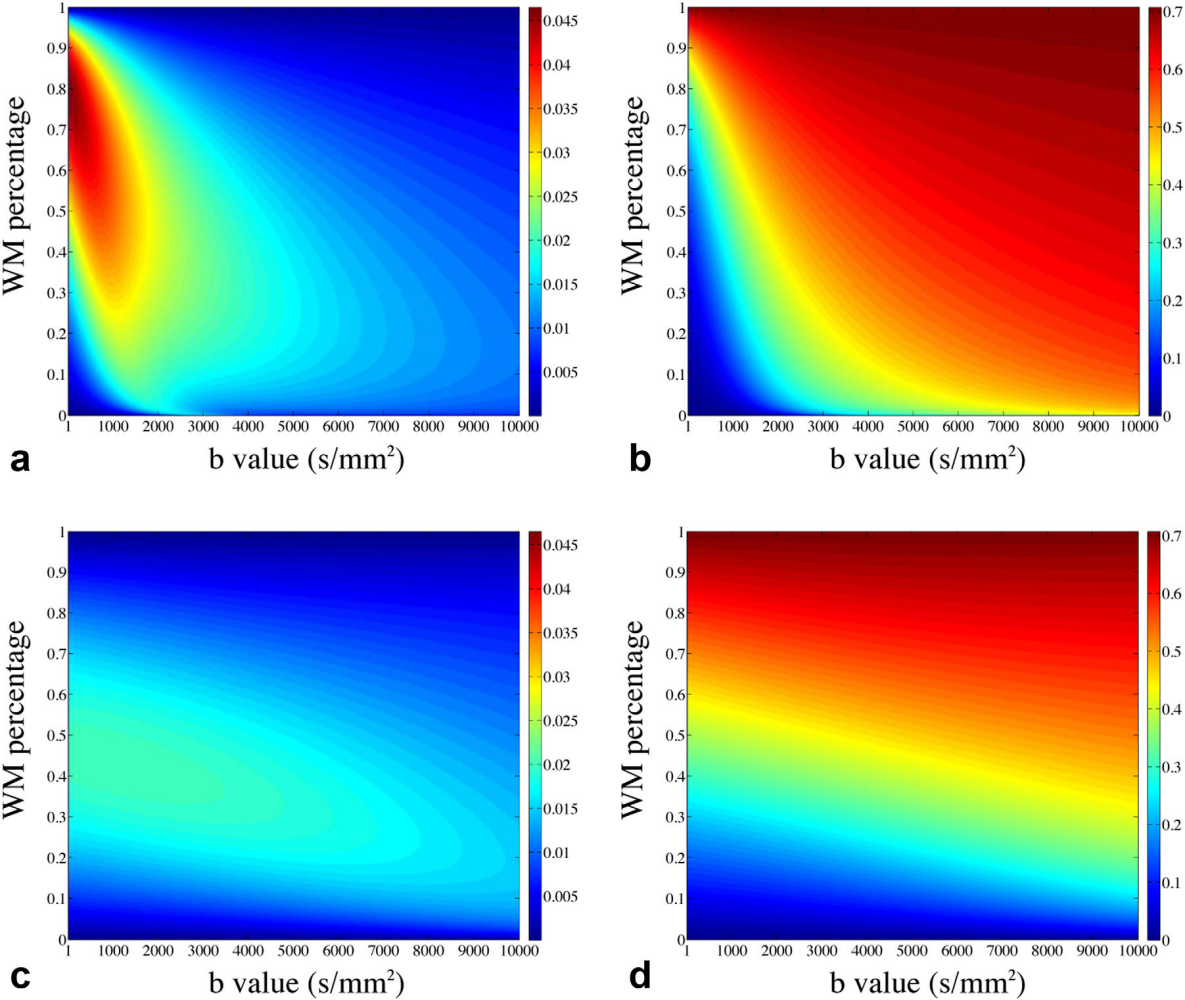
Simulation of FA errors caused by slab crosstalk. (**a**, **b**) Simulations based on a WM/CSF partial volume model: (a) the absolute FA errors (differences of FA values between slab boundary and slab center) as a function of WM percentage and b value and (b) the absolute FA values at slab center. (**c**, **d**) Simulations from a WM/GM partial volume model: (c) the absolute FA errors as a function of WM percentage and b value and (d) the absolute FA values at slab center.

**Figure 4:**
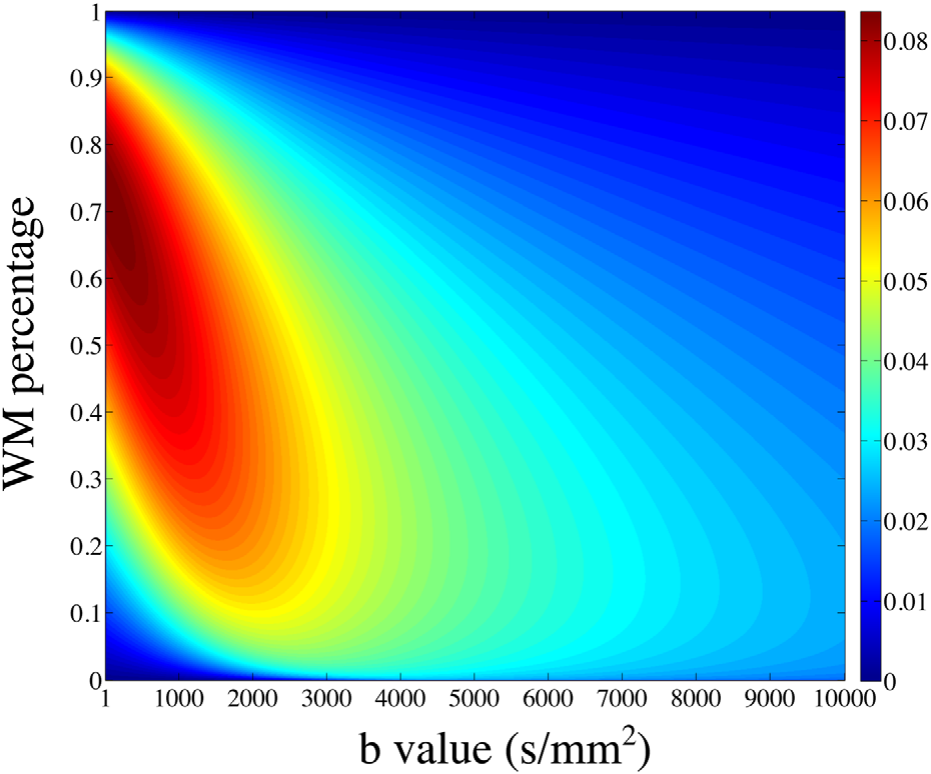
Relative MD errors as a function of WM percentage and b value simulated with WM/CSF partial volume.

Figure 3 presents the results of FA simulation from the partial volume mode. The absolute errors depend on the partial volume ratio of the tissues and the b values used in the simulation. For WM/CSF partial volume (Fig. 3a and 3b), the absolute FA error approaches its maximum value of ∼0.045 with WM percentage of ∼7% and b value smaller than 1000 s/mm^2^. For WM/GM partial volume (Fig. 3c and 3d), the maximal absolute FA error is ∼0.02. In both cases, the FA errors decrease when the b value goes up, as more isotropic‐diffusion compartments (GM and CSF) are suppressed due to their higher diffusion coefficients. Figure 4 shows the relative errors for MD from WM/CSF partial volume, which are on the order of a few percent with a maximum value of ∼8%. For WM/GM partial volume, the relative errors for MD are negligible (image not shown). Importantly, these simulations of partial volume effects demonstrate that slab profile effects in voxels with partial volume cannot be trivially removed by normalization during the calculation of diffusion parameters.

### Proposed Correction

The simulations presented in the previous sections demonstrate that slab‐boundary artifacts depend on the details of the tissue environment, including relaxation times and field inhomogeneity. Furthermore, these effects cannot be assumed to be removed by normalization with a b = 0 image in the likely case of tissue partial volume. Finally, these effects are considerably worse in the SNR‐optimal regimen of 1–2 s. The accuracy of 3D multislab dMRI thus depends on finding a method for reducing the impact of these artifacts on quantified diffusion values. It is useful to begin by reviewing slab aliasing and the previously proposed PEN method 34. We then present a new method based on PEN that aims to deal with the additional slab crosstalk issues raised by the simulations above.

The PEN method treats the 3D multislab acquisition as a linear encoding process, which can be expressed as

(2) PFCSu=d,

where *u* is the unknown 3D image, *S* = ${(s}_{1},s_{2},\ldots s_{N_{slab}})$ describes the slab profiles, *C* describes the coil sensitivities, *F* is the Fourier transform operator, *P* captures the k‐space sampling trajectory, and *d* is the acquired multislab k‐space data. This is very similar to the general parallel imaging model, except that here the image is weighted by both coil sensitivity and slab profile. Aliasing artifacts arise as a result of undersampling in the slice direction, where we here consider the effect of magnetization excited by a given pulse throughout the entire brain volume.

The quality of PEN reconstruction is critically dependent on the ability to estimate the slab profiles accurately. As shown in Figure 2, the slab crosstalk is affected by multiple factors, making it difficult to estimate slab profiles accurately using Bloch simulation. An alternative approach is to measure slab profiles from a calibration scan, which is a multislab scan at the same slab locations but oversampled in the slice direction to minimize aliasing. The slab images were first zero‐padded to the full FOV, and then a combined volume was generated by sum‐of‐squares of all the zero‐padded slabs. Finally, the slab profile was estimated by normalizing each of the zero‐padded slab images by this combined single‐slab image 34. This method will not increase the scan time significantly, but the reconstructed images will be modulated by the sum‐of‐squares of the slab profiles 34, 35, which could be relatively flat in the slice direction with a long TR (∼4 s) but would result in significant signal variation with a short TR (∼2 s).

As originally proposed, the PEN method relies on the assumption that slab profile estimates are accurate, which is increasingly difficult to ensure at short TR. To overcome this problem, we propose a new nonlinear reconstruction that jointly estimates the slab profile and the image. In this method, both slab profile and image are treated as unknowns *x* = [*u*,*S*]^*T*^, which should satisfy

E(x)=d

(3) $$E\left(x\right)=\left(PFCs_{1}u,PFCs_{2}u,\ldots ,PFCs_{N_{slab}}u\right),$$

where E is a nonlinear operator mapping image, slab profile, and coil sensitivity to the acquired data. This poses the 3D multislab reconstruction as a nonlinear inversion problem, which we refer as nonlinear inversion for slab profile encoding (NPEN). As shown previously 41, 42, 43, 44, this problem can be solved using an iteratively regularized Gauss–Newton algorithm 45, 46, 47. In step *n*, Equation (3) is first linearized around *x_n_*:

(4) $$E^{'}\left(x_{n}\right){\Delta x}_{n}=d-E\left(x_{n}\right),$$

where *E′*(*x_n_*) is the Fréchet derivative of *E* at the current guess *x_n_*, Δ*x_n_* is the update, and the new guess can be obtained by *x_n_*_+1_ = *x_n_* + Δ*x_n_*. The linear problem described by Equation (4) is then solved by the following Tikhonov regularized minimization:

(5) $$\min{∥E^{'}\left(x_{n}\right){\Delta x}_{n}-\left(d-E\left(x_{n}\right)\right)∥}^{2}+\alpha_{n}{∥x_{n}+\Delta x-x_{0}∥}^{2},$$

where the first term is the data fidelity term and the second term is the regularization term. *x*_0_ is an initial guess and *α_n_* is a regularization parameter, which is reduced in each step to benefit from the robustness at the initial steps of iterations (i.e., gradient–descent‐like) and the fast convergence when *x* is close to the solution (i.e., Gauss–Newton‐like). Equation (5) can be solved with the conjugate gradient algorithm.

As reported previously 41, 43, more accurate estimation of the solution to Equation (3) can be achieved by adding further constraints in the reconstruction. In this study, we considered two constraints. First, the slab profile was constrained to be smooth (in‐plane), which is similar to the polynomial fitting process in the slab profile estimation in PEN. The justification for this constraint is the observation that the slab profiles for WM, GM, and CSF are very similar (Fig. 2e). To realize this constraint, the preconditioning method described by Uecker et al 41 was applied, where *x* = [*u*,*S*]^*T*^ is replaced by *x* = [*u*,*W_S_FS*]^*T*^ and *W_S_* is a matrix used to penalize high spatial frequency *k_x_*‐*k_y_* components of *S* in each *k_z_* plane. The inverse of $W_{s}F$ is incorporated into the preconditioned *E* accordingly.

The second constraint exploits the fact that slab boundary artifacts are approximately periodic in image space. Therefore, residual artifacts would correspond to spikes in *k_z_* at known frequencies. By suppressing these spurious k‐space signals, images with reduced slab boundary artifacts can be reconstructed. This constraint was transformed as an additional penalty term in the reconstruction

(6) $$\min{∥E^{\prime }\left(x_{n}\right){\Delta x}_{n}-\left(d-E\left(x_{n}\right)\right)∥}^{2}+\alpha_{n}{∥x_{n}+\Delta x-x_{0}∥}^{2}+\beta_{n}{{∥W}_{u}Fu_{n}∥}^{2},$$

where *W_u_* is a weighting matrix with large values at the spike frequencies and small values at other frequencies such that ǁ*W_u_Fu_n_*ǁ^2^ penalizes residual slab boundary artifacts. β*_n_* is a regularization parameter controlling the strength of this constraint. For pure frequencies, *W_u_* would be a binary matrix; however, because the slab boundary artifacts are not pure frequencies, *W_u_* is instead formulated as a Gaussian weighting centered at the primary frequencies dictated by slab separation.

### Numerical Simulation

To test the reconstruction algorithm under controlled conditions, we generated realistic simulations of spin echo images based on the MNI‐Colin27 high‐resolution multimodal brain atlas (very high SNR T1‐ and T2‐weighted scans of a single subject) 48. Artificial 3D multislab data were generated based on the following pipeline. Tissue partial volume maps were estimated using FAST 49 based on the T1‐weighted images. The tissue‐specific slab profiles calculated from Bloch simulations were weighted by the partial volume maps and summed to generate the integrated slab profile. The T2‐weighted images were weighted by the integrated slab profile and Fourier‐transformed to produce k‐space data. Finally, the k‐space data were sampled in a way that is consistent with the acquisition of 11 slabs with 10 slices per slab to simulate aliasing effects. These simulated 3D multislab data were then reconstructed using the candidate methods (WA, PEN, and NPEN; details below) and compared with the ground truth (the original T2‐weighted atlas).

To compare the performance of PEN and NPEN at different TRs, we applied Bloch simulation at TR of 2 s, 4 s, and 7 s, respectively using SLR pulses designed with the same TBWP as used in the PEN work 34. The calculated slab profiles were used to generate simulation data in the way described above, and then the data was reconstructed using PEN and NPEN.

### *In Vivo* Studies

A diffusion‐weighted readout‐segmented echo planar imaging (EPI) sequence 6, 7, 50 was modified to acquire 3D multislab data. This sequence was further modified to oversample the central segment of b = 0 data by a factor of two. This oversampled b = 0 data could be used to estimate slab profiles for PEN or calculate initial guesses for NPEN, alleviating the need for a separate set of calibration data.

Data were acquired from four healthy subjects with informed consent in accordance with local ethics. For each subject, nine slabs with 10 slices per slab were acquired, and adjacent slabs were overlapped by two slices (20% overlapping), which resulted in 74 slices in the final reconstruction. k‐Space coverage of each slab was achieved using a 3D adaptation of a readout‐segmented EPI approach 9. The matrix size in each *k_z_* plane was 146 × 146, covered across five readout segments using 3/5 partial Fourier acquisition (i.e., only three segments were acquired) 51. Isotropic spatial resolution 1.5 × 1.5 × 1.5 mm^3^ was acquired with a FOV of 220 × 220 × 111 mm^3^. The same echo time (78 ms, 122 ms) and RF pulses were used as in the Bloch simulation, and TR was 2 s. The scan time for one diffusion direction was 1.3 min with central segment oversampling (the first b = 0 scan only) and 1 min without central segment oversampling (all other volumes).

Diffusion‐weighted images were acquired with b = 1000 s/mm^2^ using a Stejskal–Tanner diffusion preparation and 20 isotropically distributed diffusion encoding directions, and two b = 0 images. Only the first b = 0 data were oversampled for estimating the slab profile, which was then applied to the reconstruction of all other data (such that calibration data represents a constant overhead equivalent to a single volume scan). The total acquisition time was 22.3 min.

### Reconstruction

The reconstruction of readout‐segmented EPI data followed procedures described previously 6, 9, except that the oversampled central segments were reconstructed separately. Two sets of images were obtained from this reconstruction: high‐resolution 3D multislab images and low‐resolution 3D multislab images with doubled FOV in the slice direction, which were used as calibration images. Three slab‐boundary artifact correction methods—WA, PEN, and NPEN—were compared on simulation data and in vivo data.

In NPEN reconstruction, the initial guess for the image was set to zero. The calibration images were used to generate the initial guess for slab profiles as follows. These images contained no significant aliasing artifacts due to oversampling in the slice direction, such that the variation of signal intensity along the slice direction was dominated by slab profile, including side lobes that led to saturation effects. First, each slab image was zero‐padded to the full FOV, resulting in image I. The averaged signal for each slice was calculated as

(7) $$I_{ave}\left(z\right)=\sum_{r}\left|I\left(r,z\right)M\left(r,z\right)\right|/\sum_{r}\left|M\left(r,z\right)\right|,$$

where M is a brain mask generated by FSL's Brain Extraction Tool 52, *r* indicates in‐plane locations, and *z* indicates the locations in the slice direction. The averaged signal was then normalized by the signal magnitude at the slab center

(8) $$I_{ave}^{'}\left(z\right)=I_{ave}(z)/I_{ave}(z_{c}),$$

where *z*_*c*_ denotes the location of the center slice in the slab. Finally, the 3D slab profile estimate *S_est_* was generated by repeating $I_{ave}^{'}$ for all the in‐plane locations of $S_{est}$.

The initial regularization parameters α_0_ and *β*_0_ were set to ensure that after the first iteration, the residual ǁ*E*(*x*) − *d*ǁ was approximately 3/4 of the initial residual 43. This resulted in α_0_ = 0.2 and β_0_ = 0.4 for both simulated and in vivo data. The decay rates of α and β were set to 1.5, which performed well in the preliminary testing. To achieve better suppression of slab boundary artifacts, the decrease of β was stopped at 0.03.

PEN reconstruction was implemented as described by Van et al 34. The slab profile was estimated from the calibration images, in which the single slab image was divided by the sum‐of‐squares of all slab images. A 2D low‐pass Hamming filter was applied in k‐space to reduce noise in the estimated slab profiles. In WA reconstruction, a one‐dimensional (1D) Fermi filter function was applied to each slab by multiplication in the spatial domain to reduce the signal from the edge slices with aliasing, and then the weighted slab images were averaged to generate a 3D single slab image 21, 34.

Performance of different methods on the simulation data was evaluated using root‐mean‐square‐error (RMSE) and difference maps from the ground truth image. To assess the reconstruction performance on different tissue types, RMSE and difference maps were also calculated for WM, GM, and CSF separately using binary segmentation maps generated from partial volume maps with a threshold of 0.8.

For the in vivo data, eddy current correction was performed in FSL using the first b = 0 volume as a reference, after which DTI fitting was applied to generate diffusion parameter maps using FSL's diffusion toolbox 53.

## RESULTS

### Reconstruction with Simulation Data

**Figure 5:**
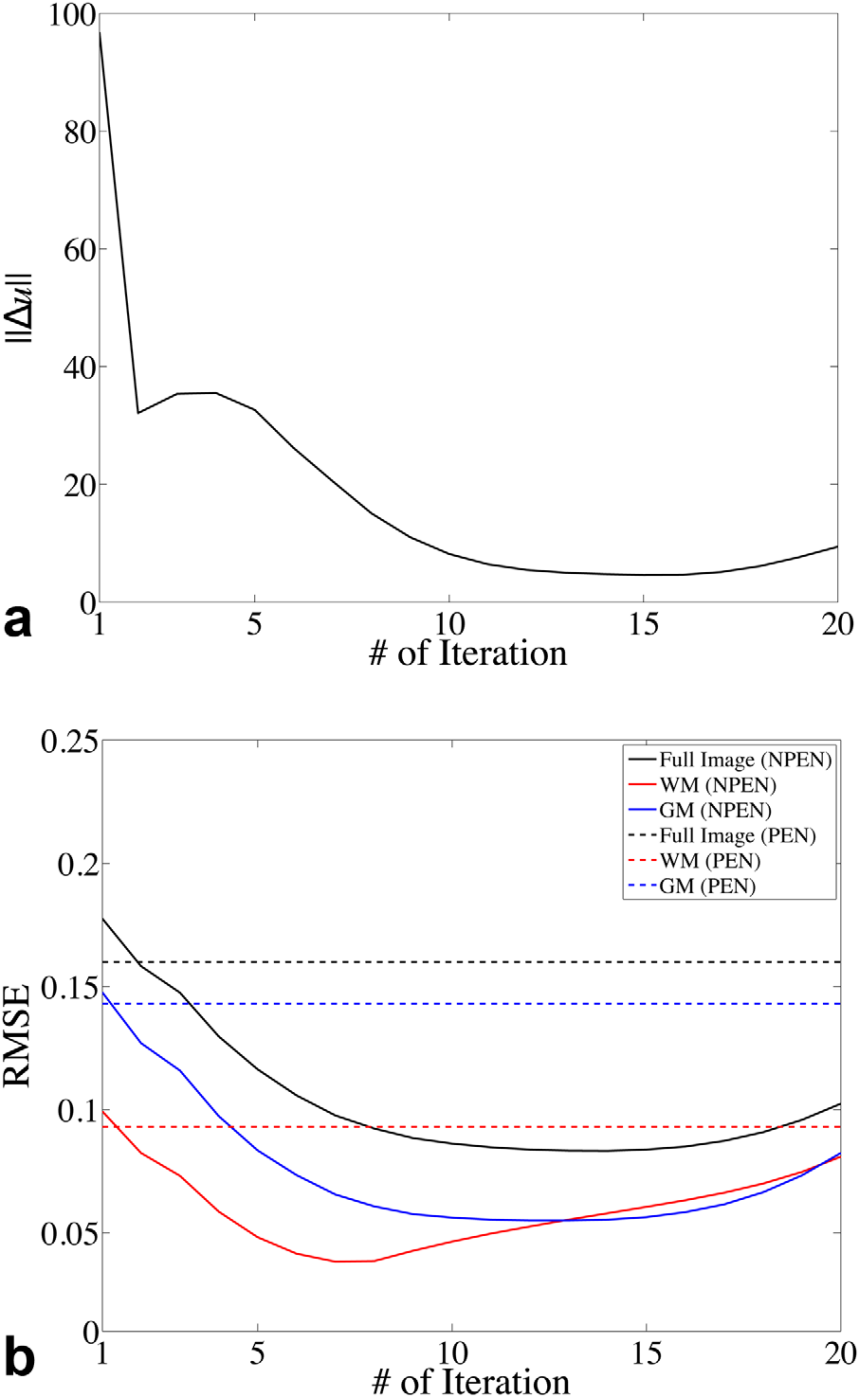
(**a**) Image update at each iteration. (**b**) RMSE for the full image (black), WM (red), and GM (blue) calculated between the reconstructed image and the ground truth at each iteration. Solid lines show the RMSE of NPEN reconstruction; dashed lines indicate the RMSE of PEN reconstruction.

Ideally, we would like to use the minimal RMSE as the stopping criteria of the iteration. However, for in vivo scans, there is no ground truth image for RMSE calculation, so we need to find another stopping criteria. The RMSEs and image update (ǁΔ*u*ǁ) from the atlas‐based simulation reconstructions are shown in Figure 5. After the first three iterations, ǁΔ*u*ǁ decreases toward its minimum and increases afterward (Fig. 5a). The iteration number corresponding to the minimum ǁΔ*u*ǁ also approximately corresponds to the lowest RMSE for the full image (Fig. 5b). This suggests that a stopping criterion based on ǁΔ*u*ǁ would be appropriate in practice (when the ground truth image is not available to calculate RMSE). However, as shown in Figure 5b, the iteration numbers corresponding to the minimal RMSE of the full image, WM, and GM are different, which probably stems from the T1 dependence of the slab profile. To achieve the best reconstruction of WM, the iteration should be stopped before the minimal RMSE of the full image, or the minimal ǁΔ*u*ǁ in practice. Therefore, in this study, the iteration was stopped with the smallest image update ǁΔ*u*ǁ, and the best reconstruction for WM was retrospectively chosen based on visual inspection. Although the final reconstruction is determined by visual inspection, the stopping criteria based on ǁΔ*u*ǁ can limit the number of iterations and reduce the computation time.

**Figure 6:**
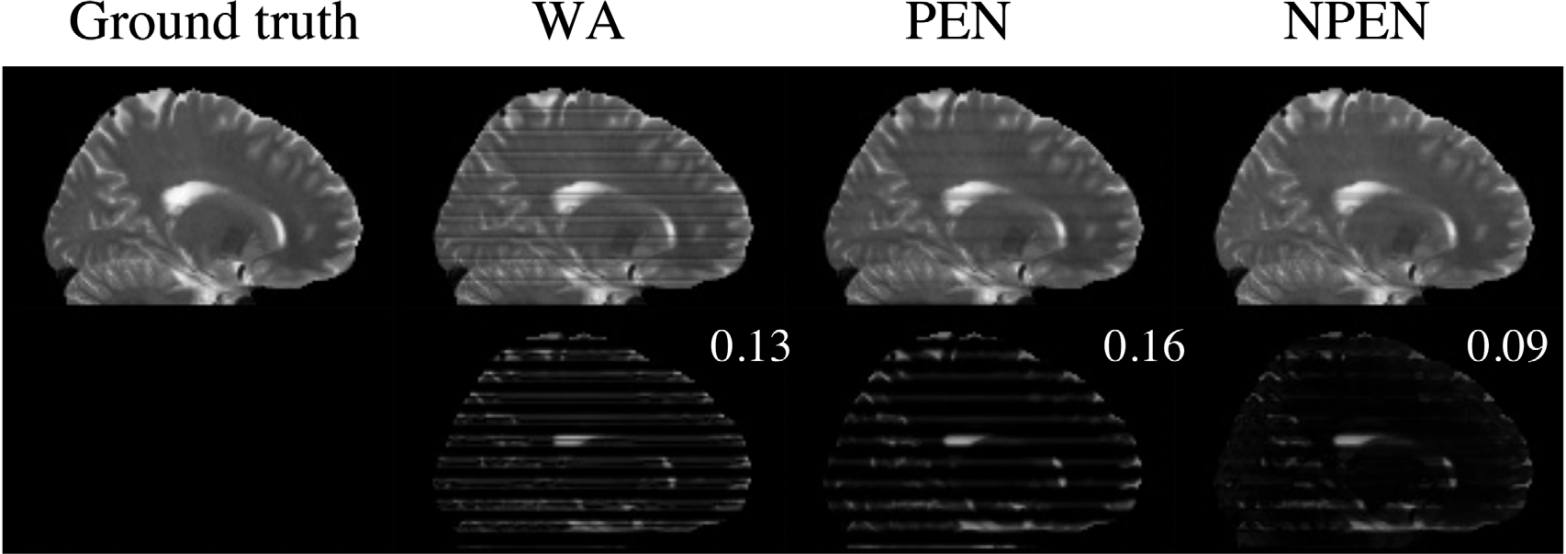
Reconstruction results from simulated data. Upper row: ground truth image and images reconstructed by WA, PEN, and NPEN. Lower row: difference maps between the reconstructed images and the ground truth; RMSE is shown in the upper right‐hand corner. The difference map is rescaled for better depiction of the error distributions.

Figure 6 shows the correction results on simulated data. WA and PEN results still retained strong artifacts at slab boundaries, whereas the NPEN result shows significantly reduced artifact level, revealed by smaller RMSE and minor residual artifacts in the difference map. It is worth noting that, according to the literature, PEN has a much lower artifact level 34, which is due to the use of longer TR corresponding to less T1 saturation but which results in lower SNR efficiency and longer scan time.

**Figure 7:**
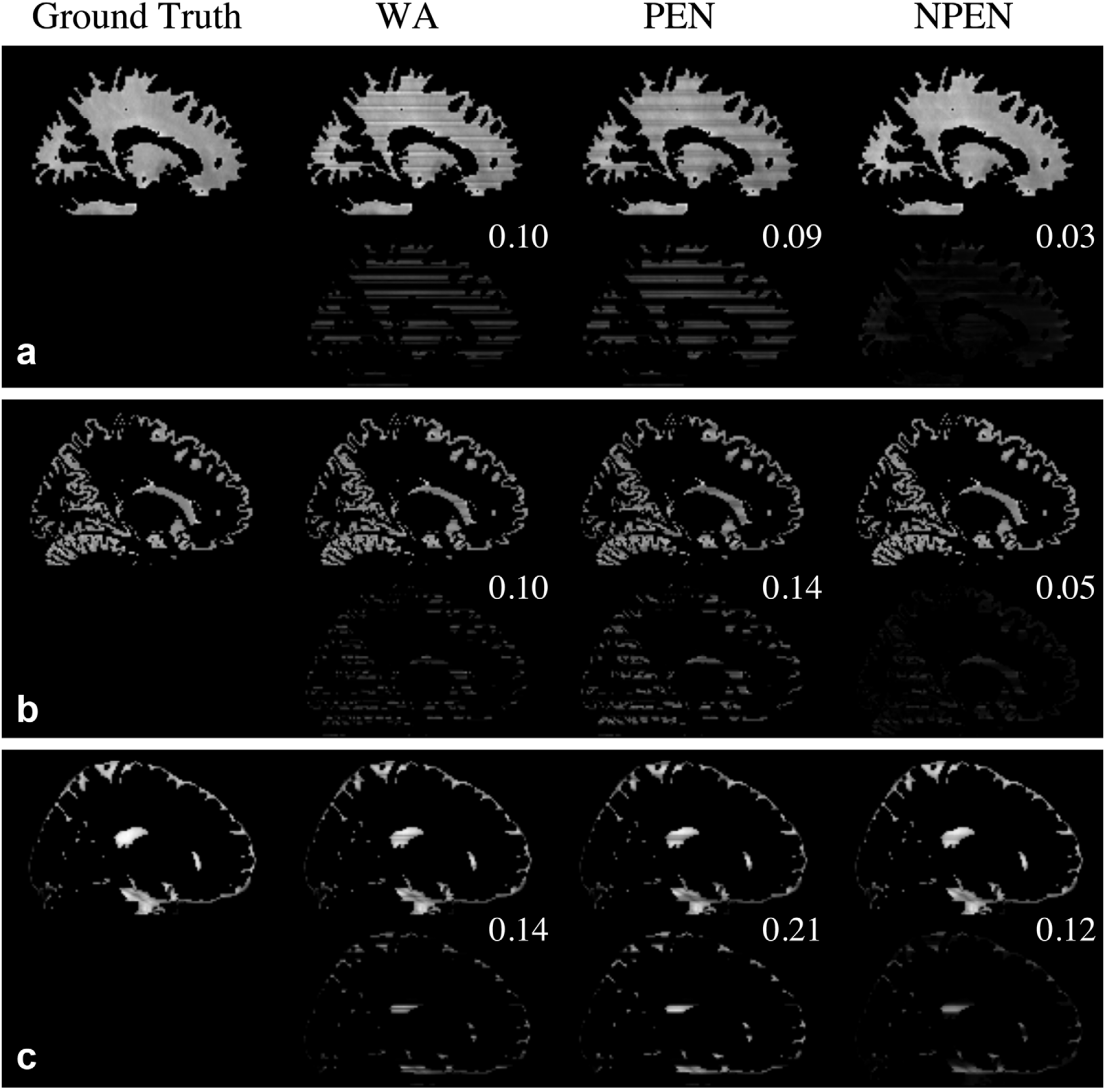
Tissue‐specific comparison of the reconstruction results from simulation data for (**a**) WM, (**b**) GM, and (**c**) CSF. For each tissue type, the upper row shows the reconstruction results, whereas the lower row shows the difference maps between the reconstructed images and the ground truth. RMSE is shown in the upper right‐hand corner of the difference map. The difference map is rescaled for better depiction of the error distributions.

Figure 7 shows the tissue‐specific comparison between three reconstruction methods. As shown in Figure 7a, the artifacts in WM have been mostly removed by NPEN, which results in an RMSE of 0.03, whereas WA and PEN results retain high artifact levels, with an RMSE of 0.1 and 0.09, respectively. The GM results (Fig. 7b) also suggest that NPEN performs better than WA and PEN. Figure 7c shows the CSF results, where none of the three methods provided a satisfactory correction, but NPEN still yielded the lowest RMSE. The tissue‐specific comparison shows that most residual artifacts in NPEN are located in CSF region, which is not problematic for dMRI.

**Figure 8:**
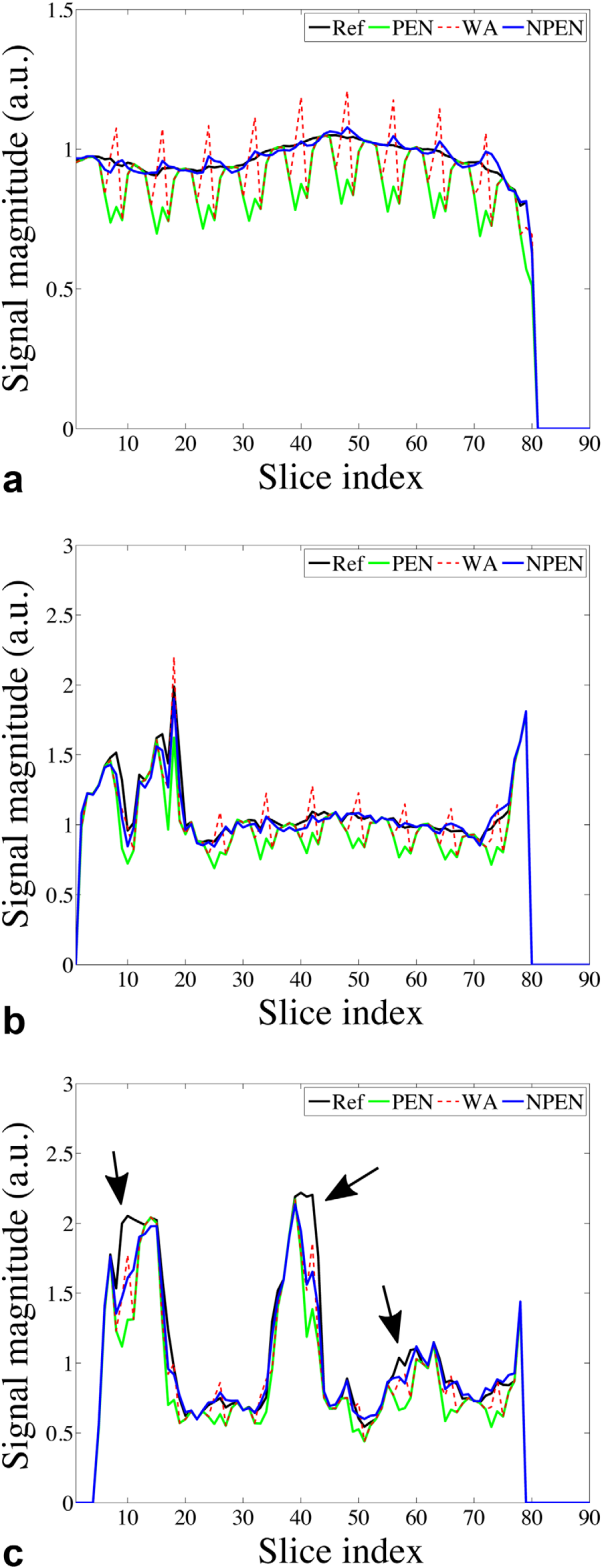
(**a**) Averaged WM signal at each slice. (**b**) Signal profile along a line passing through left putamen in the slice direction. (**c**) Signal profile along a line passing through the left ventricle in the slice direction. Black arrows indicate residual slab boundary artefacts in CSF regions.

For a clearer comparison, the WM signal was averaged in each slice and is shown in Figure 8a. NPEN reduced the signal drops at slab boundaries and generated a signal profile similar to the ground truth, whereas signal variation along the slice direction was still strong in WA and PEN. A 1D signal profile in the slice direction passing through the left putamen is shown in Figure 8b; NPEN reconstruction provided the best approximation to the ground truth. Figure 8c shows another 1D signal profile in the slice direction passing through the left ventricle, which was chosen as a worst case scenario for NPEN reconstruction. NPEN exhibited large errors around slice 10, slice 40, and slice 58 (Fig. 8c, black arrows), which corresponded to CSF regions; however, in non‐CSF regions, NPEN matched the ground truth nicely. In comparison, the WA and PEN results differed more from the ground truth in either CSF region or non‐CSF region.

**Figure 9:**
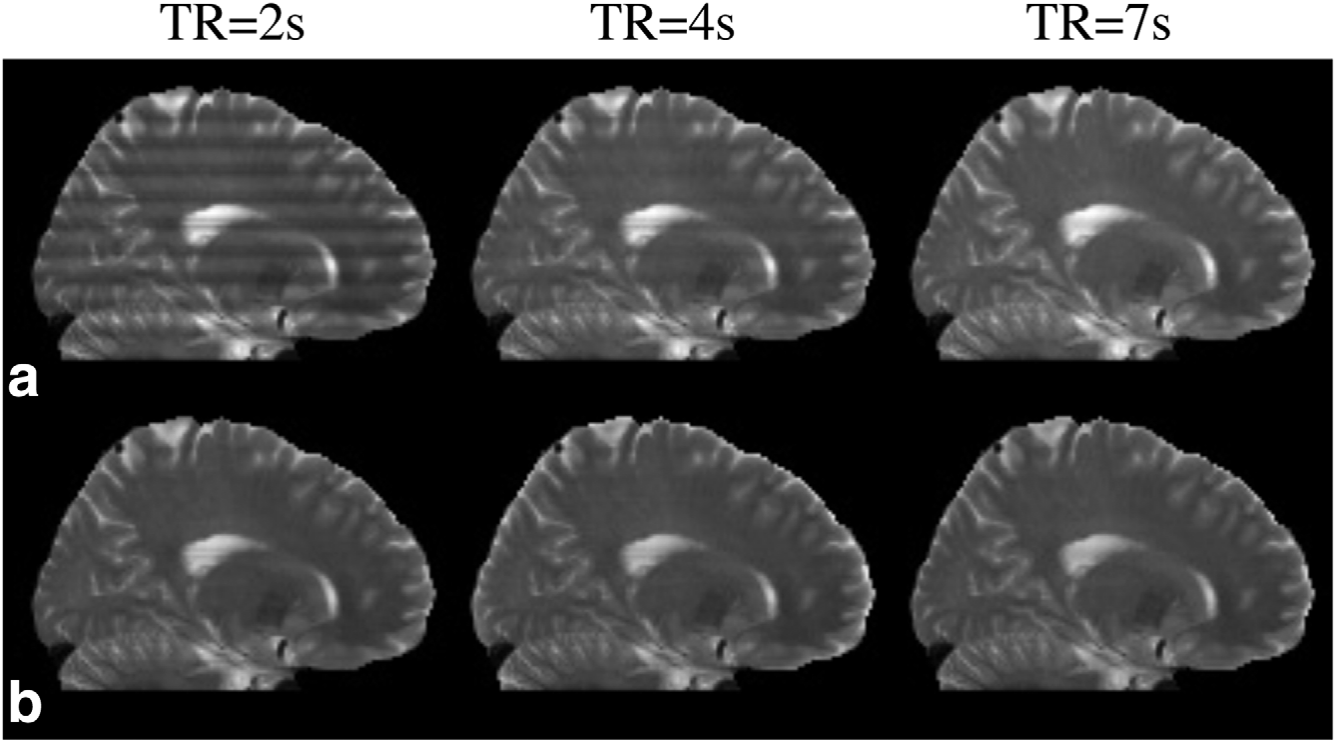
PEN (**a**) and NPEN (**b**) reconstruction at different TR.

Figure 9 shows results of PEN and NPEN at different TR. It can be seen that the residual artifacts in PEN reconstruction at short TR (2 s) are very strong but can be reduced at longer TRs (4 and 7 s). NPEN can effectively reduce slab boundary artifacts regardless of TR values.

### Reconstruction with In Vivo Data

**Figure 10:**
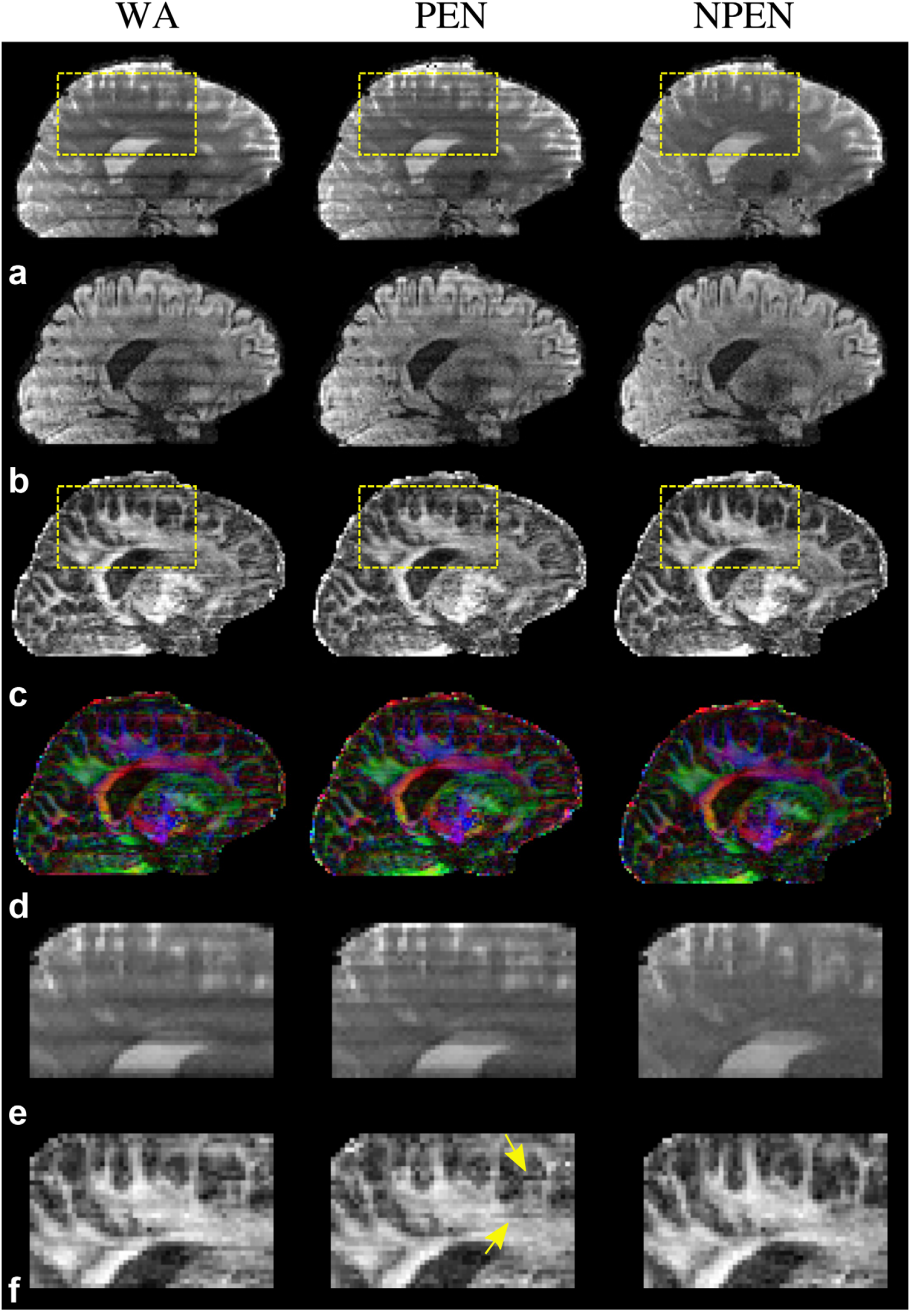
Comparison of three reconstruction methods based on in vivo data. (**a**) b = 0 image. (**b**) DWI image (b = 1000 s/mm^2^). (**c**) FA maps. (**d**) Color‐coded maps of the principal eigenvector (red, right–left; green, anterior–posterior; blue, superior–inferior). (**e**, **f**) Zoomed‐in regions specified by the yellow rectangles in panels a and c, respectively.

Figure 10 compares the reconstruction methods on in vivo data. As shown in b = 0 images (Fig. 10a and 10e) and diffusion‐weighted images (Fig. 10b), WA and PEN still contained strong slab boundary artifacts, whereas NPEN effectively reduced the artifact level with only minor residual artifacts visible in CSF regions. As shown in the FA maps (Fig. 10c) and color‐coded maps of the principal eigenvector (Fig. 10d), WA still had strong artifacts at slab boundaries even after normalization by the b = 0 image. The PEN result showed reduced artifacts in the FA map compared with WA but retained some residual errors, as indicated by yellow arrows in the zoomed‐in FA map (Fig. 10f). Given that the primary goal of PEN was to remove aliasing, this suggests that the effect of aliasing artifacts in WA reconstruction is significant. NPEN achieves the best results, with almost no visible artifact, suggesting that NPEN is effective at removing slab boundary artifacts associated with the RF profile, aliasing and saturation. Reconstructions based on data acquired from a different subject are shown in Supporting Figure S3, demonstrating similar performance of the different methods.

## DISCUSSION

Several methods have been proposed to correct slab boundary artifacts in dMRI, including the weighted average and the PEN methods considered here. Weighted average avoids aliasing at slab boundaries by oversampling along the slab direction. The goal of PEN is to remove aliasing artifacts, with no attempt to address the saturation effects. Thus, PEN works well under the condition that the sum‐of‐squares image used in slab profile estimation has a relatively flat signal profile. If not, residual signal variation remains in the reconstructed images and can propagate into diffusion parameter maps such as MD and FA. Our NPEN method extends PEN to deal with both aliasing and slab crosstalk, which enables correction of slab boundary artifacts under conditions of strong T1 saturation due to short TR (1–2 s) associated with optimal SNR efficiency.

The conventional coupling between FOV and TR is lessened in multislab acquisition. Encoding of slices through a combination of slab excitation and within‐slab phase encoding confers flexibility in achieving a desired coverage and TR without major impact to scan time. With short TR, we can only accommodate a small number of excitation periods. At longer TR, we can achieve the same coverage by increasing the number of slabs while reducing the within‐slab phase encoding, avoiding significant increase in total scan time. Moreover, one could also deploy simultaneous multislab acceleration 54 to increase coverage without requiring more excitations per TR. As such, the need for longer TR to improve reconstruction in PEN and WA does not in itself require longer scan time; and conversely, the key benefit conferred by NPEN in enabling short TR is increased SNR efficiency, not shorter scan times.

Nevertheless, one drawback to all slab boundary corrections is a reduction in scan time efficiency. At its most efficient, the number of *k_z_* phase encodings would simply be equal to the number of reconstructed z slices (although this would require a perfect rectangular slab profile, which cannot be achieved in practice). We can define scan time efficiency in terms of the fractional increase in scan time beyond this idealized case. All slab boundary corrections have reduced scan time efficiency due to additional measurements associated with either 1) deliberate overlapping of adjacent slab FOVs in the transition bands or 2) oversampling of excited slab FOVs into their transition regions. WA uses both overlapping and oversampling, with the original implementation requiring 0.4 times more *k_z_* encodings than reconstructed slices, or 71.4% scan time efficiency 21. Both PEN and NPEN overlap slab FOVs by ∼20% and also incur a fixed overhead to acquire calibration data (here, equivalent to acquiring one additional volume). Hence, our protocol with two b = 0 and 20 diffusion‐weighted volumes had a 79.7% scan time efficiency.

Two constraints were applied in NPEN. The in‐plane smoothness constraint imposed on the slab profile was used to improve the conditioning of the problem. This constraint seemed reasonable given that Bloch simulations of the slab profiles for WM, GM, and CSF are quite similar and B0/B1 profiles vary smoothly over space, although WM and CSF profiles do exhibit consistent differences (Fig. 2e). Nevertheless, the tissue‐specific comparison (Fig. 7) suggests the artifacts in WM and GM components are nicely corrected, with greatest residual artifacts being in CSF, which is only of interest in areas of large partial volume. The second constraint was used to suppress the spurious frequency components corresponding to slab boundary artifacts. A similar approach was adopted by Engstrom et al 27, who used a band‐pass filter in the estimation of a 1D banding function, which was applied to correct slab boundary artifacts. The main difference between the two approaches is that NPEN allows the spatial variation of the slab profile within each slice plane, capturing the effects caused by different tissue types and B0 inhomogeneity, whereas the 1D banding function used in the previous method can only provide a global correction. The effects of these constraints on NPEN reconstruction are demonstrated in Supporting Figure S4. The reconstruction RMSE increases after a certain number of iterations. This likely stems from the overfitting, which may occur due to the high complexity of the nonlinear reconstruction model. In the reconstruction, the algorithm tries to improve the fitting of the unknowns based on the reconstruction model. However, after a certain point, the improvement on the fitting of the unknowns comes at the expense of increased errors, leading to inflated RMSE. As the RMSE curves for WM and GM are different, in some cases it is possible the error in GM decreases while the error in WM increases; however, as shown in Figure 5, this is not a significant effect, which is not clearly visible in our results.

In the PEN method, subject motion could be an outstanding problem as the slab profile estimated from the calibration scan might be misaligned with the images acquired later, especially for DTI where a large number of volumes are required and scan time is long. In comparison, NPEN is a data‐driven method, in which the calibration image is only used for initialization and its effect is reduced during the iterative reconstruction, so NPEN is expected to be less sensitive to subject motion.

The long scan times (1 min per volume) used in the data acquisition are dictated by the use of readout segmentation and are the trade‐off associated with reduced distortion and T_2_* blurring. It should be noted that acquisition with single‐shot per *k_z_* plane (with equivalent distortion and T_2_* blurring to 2D single‐shot) can achieve ∼20 s per volume, which is about 70% longer than a conventional 2D single‐shot EPI due to the need for navigator echoes and slab overlap, but would still have about 50% higher SNR efficiency.

A drawback of NPEN is the computational cost. This is in part due to the operations on large matrix and iterative nature of the method. The computation time for all diffusion directions was ∼1 h using MATLAB (2014a; MathWorks, Natick, Massachusetts, USA) on a distributed computing cluster. The cluster consisted of 19 computers, eight of which had AMD Opteron Processors 6328 (32 cores, 3.2GHz) and 512 GB RAM, the others of which had Intel Xeon Processors E5640 (16 cores, 2.67GHz) and 64 GB RAM. It should be noted that the computation time could be definitely reduced with optimized C/C++ implementation.

The NPEN method was investigated here in the context of diffusion measurements. However, this method may be applicable to other MRI applications using 3D multislab acquisition, such as 3D fast spin echo imaging and time‐of‐flight angiography.

## CONCLUSIONS

We present a new approach to reduce the slab boundary artifacts in 3D multislab dMRI. Slab boundary artifacts were evaluated using Bloch simulations for a range of tissue properties and acquisition parameters, with particular interest on the potential for these artifacts to bias estimates of diffusion‐based quantification (e.g., FA and MD). The presented reconstruction method, NPEN, can correct both the aliasing artifacts and signal variation caused by slab crosstalk even at short TR (∼2 s). Reconstruction results on simulated and in vivo data demonstrate that the proposed method improves upon previous methods, including the original PEN technique.

## Supporting information


**Supporting Figure S1.** (**a**, **b**) Waveforms of the excitation pulse (a) and refocusing pulse (b) used in the simulations and in vivo scans. (**c**) Simulated signal profile with one slab (TR = 2 s).
**Supporting Figure S2.** WM signal profile of one slab simulated at different off‐resonance frequencies.
**Supporting Figure S3.** Coronal slice of the reconstruction results based on the data acquired from a different subject. (**a**) b = 0 image. (**b**) DWI image (b = 1000 s/mm^2^). (**c**) FA maps. (**d**) Color‐coded maps of the principal eigenvector (red, right‐left; green, anterior–posterior; blue, superior–inferior). (**e**, **f**) Zoomed‐in regions specified by the yellow rectangles in panels a and c, respectively.
**Supporting Figure S4.** Reconstruction results using iteratively regularized Gauss–Newton method. (**a**) Without additional constraints. (**b**) With in‐plane smoothness constraints on slab profile. (**c**) With frequency constraints on image. (**d**) With both in‐plane smoothness constraints on slab profile and frequency constraints on image. All other reconstruction parameters (e.g., number of iterations, regularization parameters) are the same in the reconstructions.
