# Supplementary material for: Reducing slab boundary artifacts in three‐dimensional multislab diffusion MRI using nonlinear inversion for slab profile encoding (NPEN)
Source: Magn Reson Med. 2015 Oct 28;76(4):1183–95. doi: 10.1002/mrm.26027 (PMC4854328; doi:10.1002/mrm.26027)
Supplement: Supplementary file 1 — Supporting Figure S1. (a, b) Waveforms of the excitation pulse (a) and refocusing pulse (b) used in the simulations and in vivo scans. (c) Simulated signal profile with one slab (TR = 2 s). Supporting Figure S2. WM signal profile of one slab simulated at different off‐resonance frequencies. Supporting Figure S3. Coronal slice of the reconstruction results based on the data acquired from a different subject. (a) b = 0 image. (b) DWI image (b = 1000 s/mm2). (c) FA maps. (d) Color‐coded maps of the principal eigenvector (red, right‐left; green, anterior–posterior; blue, superior–inferior). (e, f) Zoomed‐in regions specified by the yellow rectangles in panels a and c, respectively. Supporting Figure S4. Reconstruction results using iteratively regularized Gauss–Newton method. (a) Without additional constraints. (b) With in‐plane smoothness constraints on slab profile. (c) With frequency constraints on image. (d) With both in‐plane smoothness constraints on slab profile and frequency constraints on image. All other reconstruction parameters (e.g., number of iterations, regularization parameters) are the same in the reconstructions. [file MRM-76-1183-s001.pdf]

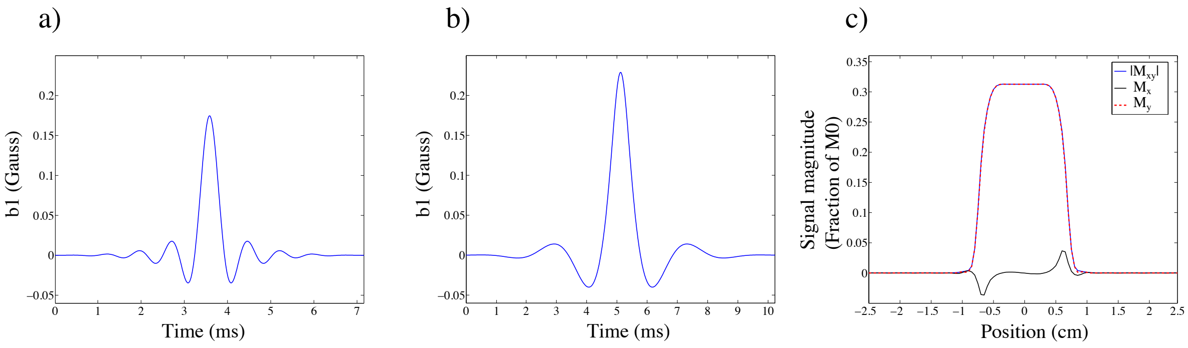

Supporting FIG. S1. The waveforms of the excitation pulse (a) and the refocusing pulse (b) used in the simulation and in vivo scans. (c). The simulated signal profile with 1 slab, 2s TR.

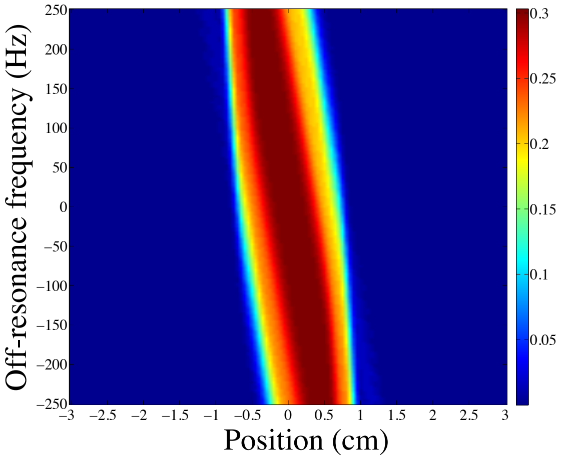

Supporting FIG. S2. WM signal profile of one slab simulated at different off-resonance frequencies.

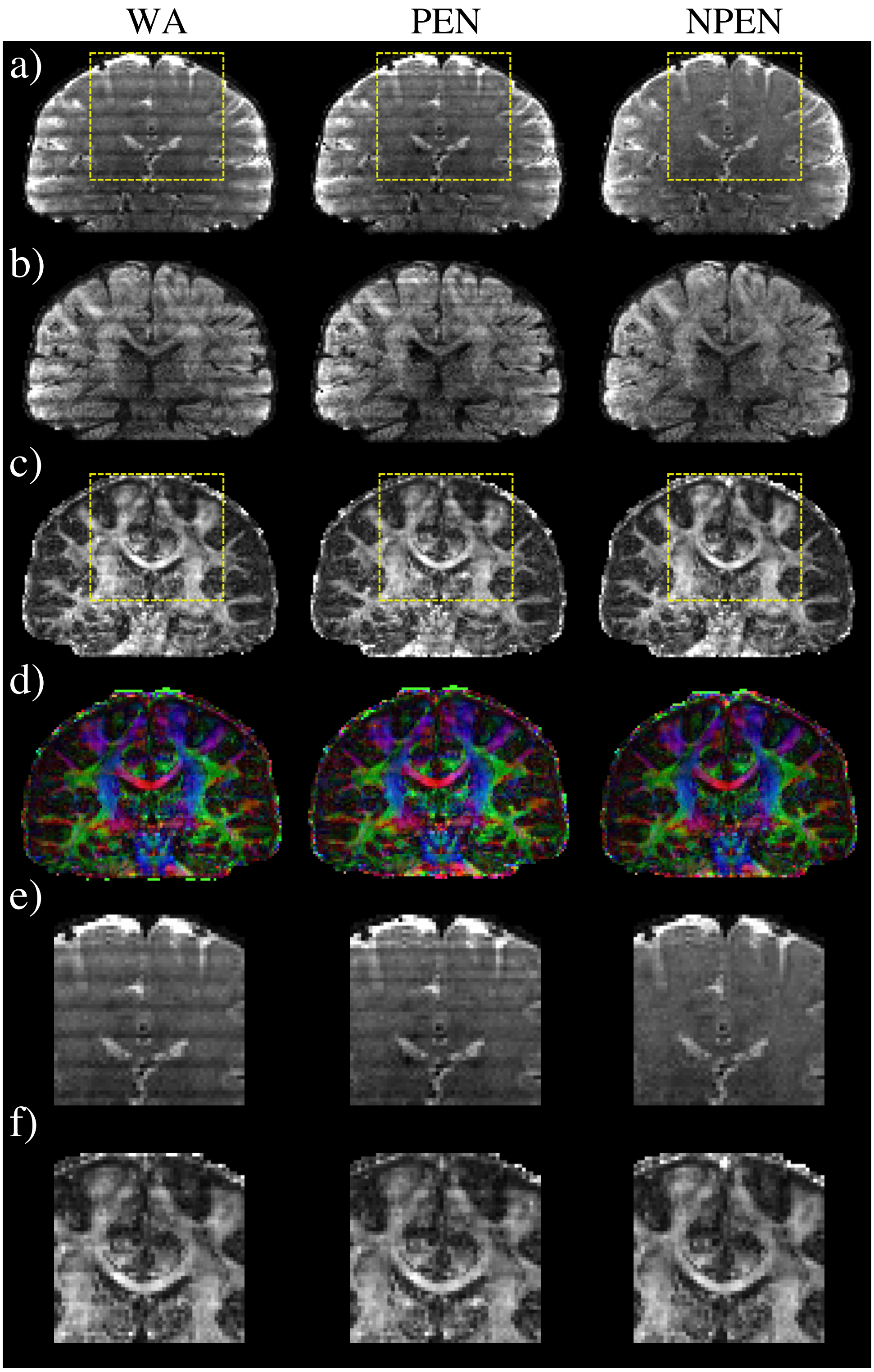

Supporting FIG. S3. A coronal slice of the reconstruction results based on the data acquired from a different subject. (a) b = 0 image. (b) DWI image (b=1000 s/mm^2). (c) FA maps. (d) Color-coded maps of the principal eigenvector (red: right-left, green: anterior-posterior, blue: superior-inferior). (e) and (f) are the zoomed-in regions specified by the yellow rectangles in (a) and (c) respectively.

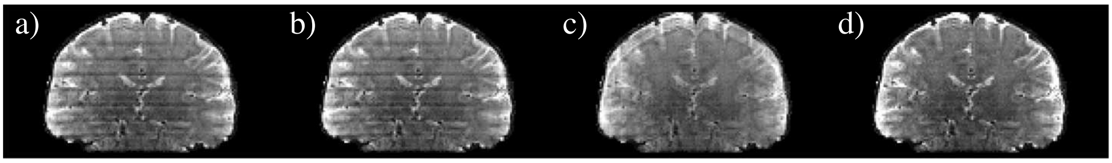

Supporting FIG. S4. Reconstruction results using iteratively regularised Gauss-Newton method: (a) without additional constraints, (b) with in-plane smoothness constraints on slab profile, (c) with frequency constraints on image, (d) with both in-plane smoothness constraints on slab profile and frequency constraints on image. All other reconstruction parameters (e.g. number of iterations, regularisation parameters) are the same in the reconstructions.
